# Asthma inflammatory phenotypes on four continents: most asthma is non-eosinophilic

**DOI:** 10.1093/ije/dyac173

**Published:** 2022-08-30

**Authors:** Lucy Pembrey, Collin Brooks, Harriet Mpairwe, Camila A Figueiredo, Aida Y Oviedo, Martha Chico, Hajar Ali, Irene Nambuya, Pius Tumwesige, Steven Robertson, Charlotte E Rutter, Karin van Veldhoven, Susan Ring, Mauricio L Barreto, Philip J Cooper, John Henderson, Alvaro A Cruz, Jeroen Douwes, Neil Pearce, the WASP Study Group, Neil Pearce, Lucy Pembrey, Steven Robertson, Karin van Veldhoven, Charlotte E Rutter, Sinead Langan, Sarah Thorne, Donna Davoren, John Henderson, Susan Ring, Elizabeth Brierley, Sophie Fitzgibbon, Simon Scoltock, Amanda Hill, Alvaro Cruz, Camila Figueiredo, Mauricio Barreto, Cinthia Vila Nova Santana, Gabriela Pimentel, Gilvaneide Lima, Valmar Bião Lima, Jamille Fernandes, Tamires Cana Brasil Carneiro, Candace Andrade, Gerson Queiroz, Anaque Pires, Milca Silva, Jéssica Cerqueira, Philip Cooper, Martha Chico, Cristina Ardura-Garcia, Araceli Falcones, Aida Y Oviedo, Andrea Zambrano, Jeroen Douwes, Collin Brooks, Hajar Ali, Jeroen Burmanje, Harriet Mpairwe, Irene Nambuya, Pius Tumwesige, Milly Namutebi, Marble Nnaluwooza, Mike Mukasa

**Affiliations:** Department of Medical Statistics, London School of Hygiene & Tropical Medicine, London, UK; Centre for Public Health Research, Massey University, Wellington, New Zealand; MRC/UVRI and LSHTM Uganda Research Unit, Entebbe, Uganda; Institute of Collective Health, Federal University of Bahia, Salvador, Brazil; Fundacion Ecuatoriana Para Investigacion en Salud, Quito, Ecuador; Fundacion Ecuatoriana Para Investigacion en Salud, Quito, Ecuador; Centre for Public Health Research, Massey University, Wellington, New Zealand; MRC/UVRI and LSHTM Uganda Research Unit, Entebbe, Uganda; MRC/UVRI and LSHTM Uganda Research Unit, Entebbe, Uganda; Department of Medical Statistics, London School of Hygiene & Tropical Medicine, London, UK; Department of Medical Statistics, London School of Hygiene & Tropical Medicine, London, UK; Department of Non-communicable Disease Epidemiology, London School of Hygiene & Tropical Medicine, London, UK; Population Health Sciences, Bristol Medical School, University of Bristol, Bristol, UK; MRC Integrative Epidemiology Unit at University of Bristol, Bristol, UK; Institute of Collective Health, Federal University of Bahia, Salvador, Brazil; Center for Data and Knowledge Integration for Health (CIDACS), Fiocruz, Bahia, Brazil; Fundacion Ecuatoriana Para Investigacion en Salud, Quito, Ecuador; School of Medicine, Universidad Internacional del Ecuador, Quito, Ecuador; Institute of Infection and Immunity, St George’s University of London, London, UK; Population Health Sciences, Bristol Medical School, University of Bristol, Bristol, UK; ProAR, Federal University of Bahia, Salvador, Brazil; Institute for Health Sciences, Federal University of Bahia, Salvador, Brazil; Centre for Public Health Research, Massey University, Wellington, New Zealand; Department of Medical Statistics, London School of Hygiene & Tropical Medicine, London, UK; Centre for Public Health Research, Massey University, Wellington, New Zealand

**Keywords:** Asthma, inflammatory phenotypes, children, adolescents, sputum induction, LMIC, HIC

## Abstract

**Background:**

Most studies assessing pathophysiological heterogeneity in asthma have been conducted in high-income countries (HICs), with little known about the prevalence and characteristics of different asthma inflammatory phenotypes in low-and middle-income countries (LMICs). This study assessed sputum inflammatory phenotypes in five centres, in Brazil, Ecuador, Uganda, New Zealand (NZ) and the United Kingdom (UK).

**Methods:**

We conducted a cross-sectional study of 998 asthmatics and 356 non-asthmatics in 2016–20. All centres studied children and adolescents (age range 8–20 years), except the UK centre which involved 26–27 year-olds. Information was collected using questionnaires, clinical characterization, blood and induced sputum.

**Results:**

Of 623 asthmatics with sputum results, 39% (243) were classified as eosinophilic or mixed granulocytic, i.e. eosinophilic asthma (EA). Adjusted for age and sex, with NZ as baseline, the UK showed similar odds of EA (odds ratio 1.04, 95% confidence interval 0.37–2.94) with lower odds in the LMICs: Brazil (0.73, 0.42–1.27), Ecuador (0.40, 0.24–0.66) and Uganda (0.62, 0.37–1.04). Despite the low prevalence of neutrophilic asthma in most centres, sputum neutrophilia was increased in asthmatics and non-asthmatics in Uganda.

**Conclusions:**

This is the first time that sputum induction has been used to compare asthma inflammatory phenotypes in HICs and LMICs. Most cases were non-eosinophilic, including in settings where corticosteroid use was low. A lower prevalence of EA was observed in the LMICs than in the HICs. This has major implications for asthma prevention and management, and suggests that novel prevention strategies and therapies specifically targeting non-eosinophilic asthma are required globally.

Key Messages

- This study shows for the first time that only about one-third of asthmatics in centres in low-and-middle-income countries have eosinophilic asthma (EA).
- This study confirms previous research in high-income countries that only about one-half of asthmatics have EA.
- This has major implications for asthma prevention and management globally and highlights the need to develop new therapies, management and intervention strategies which specifically target and improve clinical outcomes in non-eosinophilic asthma.

## Introduction

Despite decades of research, knowledge of the causes and mechanisms of asthma is limited, hampering the development of effective prevention strategies.^1^ An important reason for the slow progress is that most studies do not differentiate between asthma phenotypes, despite environmental causes, pathophysiological mechanisms and optimal therapeutic interventions potentially being different for each asthma pheno/endotype.^1^

Airway eosinophilia is considered a common characteristic of asthma in high-income countries (HICs), but multiple phenotypes and endotypes have been reported,^1^^,^^2^ and in general less than one-half of cases are attributable to eosinophilic airways inflammation, with little known about the underlying causes and mechanisms of non-eosinophilic phenotypes.^3^^,^^4^ In contrast, relatively little is known about the prevalence and characteristics of asthma phenotypes in low- and middle-income countries (LMICs). Whereas asthma in LMICs appears to be largely non-atopic,^5^ or only weakly associated with atopy when compared with HICs^5^^,^^6^ (thus suggesting a limited role for airway eosinophilia/TH2-mediated inflammation), few studies in LMICs have assessed airway pathology.

Better characterization of asthma sputum inflammatory phenotypes in different settings (capitalizing on the natural variance in asthma prevalence, environmental exposures and cultural and (epi)-genetic backgrounds) is critical as it will: (i) improve understanding of the different aetiological mechanisms underlying the umbrella term ‘asthma’ (as advocated by a recent *Lancet* Commission^1^); (ii) identify specific causes and exposures; and (iii) guide the development of new therapeutic and prevention measures that are effective for all asthmatics in both HICs and LMICs. This is particularly important because non-eosinophilic asthma (NEA) is less responsive to corticosteroids,^7–9^ the current mainstay of asthma treatment.

The World ASthma Phenotypes (WASP) study is an international collaboration to investigate and characterize asthma phenotypes in HICs and LMICs (detailed rationale and protocol published elsewhere^10^). The current study tests the hypothesis that the prevalence of inflammatory asthma phenotypes differs between HICs and LMICs. Here we present findings with regards to the main four asthma inflammatory phenotypes in sputum:^11^ eosinophilic asthma (EA), involving raised eosinophil counts either without (eosinophilic) or with (mixed granulocytic) raised neutrophil counts; and non-eosinophilic asthma (NEA), involving neutrophilic airways inflammation without eosinophilia (neutrophilic) or with no apparent inflammation of the airways (paucigranulocytic). We assessed the prevalence and distribution of these asthma phenotypes, and compared their clinical characteristics in and between the different centres.

## Methods

Detailed study methods have been published elsewhere,^10^ but are briefly summarized here. The study was conducted in five centres; Bristol, UK (Avon Longitudinal Study of Parents and Children, ALSPAC^12–14^) Wellington, New Zealand, Salvador, Brazil, Quininde, Ecuador and Entebbe, Uganda (Table 1). The International Study of Asthma and Allergies in Childhood (ISAAC) found these centres to have a range of asthma prevalence levels and different environmental exposures.^15^

**Table 1:** Characteristics of centres, participants overall and asthma cases

	Brazil (Salvador)	Ecuador (Quininde)	Uganda (Entebbe)	New Zealand (Wellington)	UK (Bristol)
Study type	SCAALA cohort; new data collection and cross-sectional study in three schools	Population-based cohort; new data collection and cross-sectional study in schools	Case-control study in children recruited from schools	NZA2CS birth cohort; new data collection and cross-sectional study in schools and community	ALSPAC birth cohort study
Recruitment	244	243	257	367	243
Female (%)	161 (66%)	99 (41%)	183 (71%)	192 (52%)	177 (73%)
Age, years: mean (range)	18.5 (12.0–23.9)	11.9 (10.3–16.9)	15.5 (10.0–18.9)	14.3 (8.6–20.3)	26.0 (24.6–27.3)
Asthma cases	*n* = 204	*n *= 176	*n* = 207	*n* = 235	*n* = 176
Wheezing or whistling in the chest^a^	180 (88%)	175 (99%)	206 (99%)	191 (81%)	150 (85%)
Prior asthma diagnosis confirmed by doctor	150 (74%)	108 (61%)	141 (68%)	214 (91%)	171 (97%)
Severe asthma^a^					
Based on ISAAC	118 (66%)	79 (45%)	136 (66%)	112 (48%)	79 (45%)
Based on >12 attacks^a^	17 (8%)	0 (0%)	39 (19%)	36 (15%)	27 (15%)
Asthma medication^a^					
None	55 (27%)	99 (56%)	55 (27%)	4 (6%)	27 (15%)
ICS (preventer inhaler)	43 (21%)	6 (3%)	33 (16%)	62 (69%)	94 (53%)
Corticosteroid tablets or syrup	22 (11%)	5 (3%)	82 (40%)	8 (3%)	3 (2%)
Bronchodilator (reliever inhaler)	124 (61%)	27 (15%)	82 (40%)	210 (89%)	145 (82%)
Salbutamol tablets/syrup or aminophylline tablets/injections	3 (1%)	37 (21%)	112 (54%)	0 (0%)	0 (0%)
Other (e.g. leukotriene receptor antagonist)	2 (1%)	5 (3%)	0 (0%)	3 (1%)	2 (1%)
ACQ score (past week)	*n* = 201	*n* = 176	*n* = 168	*n* = 210	*n* = 171
Median (IQR)	0.67 (0.17–1.5)	0 (0–0)	0.67 (0–1.58)	0.67 (0.17–1.17)	0.33 (0–1)
(range)	(0–4.17)	(0–2.67)	(0–5)	(0–4)	(0–3)
Well-controlled (score < 1.5)	144 (72%)	168 (95%)	119 (71%)	170 (81%)	154 (90%)
Not well-controlled (score ≥1.5)	57 (28%)	8 (5%)	49 (29%)	40 (19%)	17 (10%)

SCAALA, Social Change, Asthma and Allergy in Latin America; NZA2CS, New Zealand Asthma and Allergy Cohort Study; ALSPAC, Avon Longitudinal Study of Parents and Children; ISAAC, International Study of Asthma and Allergies in Childhood; ICS, inhaled corticosteroids; ACQ, Asthma Control Questionnaire; IQR, interquartile range.

a In the past 12 months.

Recruitment methods differed by centre (Table 1): in four centres (Brazil,^16^ Ecuador,^16^ New Zealand^17^ and the UK^12–14^) participants were recruited from ongoing cohort studies, and in three of these (Brazil, Ecuador, New Zealand) additional recruitment was through the community (usually from surveys in schools). In Uganda, participants were recruited from a larger case-control study of asthmatics and non-asthmatics identified through a cross-sectional survey in schools.^18^ Subjects with chronic disease (except asthma) or who were pregnant were excluded from the study. Ever wheezing is the most sensitive indicator of the diagnosis of asthma.^19^ Since we are interested in current asthma*,* it was defined as wheeze or whistling in the chest and/or use of asthma medication in the past 12 months, using the ISAAC questionnaire. This ISAAC questionnaire and asthma definition have been successfully validated in various countries.^20–23^ Non-asthmatics had no history of asthma, using the same questionnaire.

The study clinic appointment was postponed if participants had an acute exacerbation of asthma or a symptomatic respiratory infection in the past 4 weeks. All participants were asked to stop taking antihistamines (5 days prior), steroid nasal sprays (7 days prior) and non-steroidal anti-inflammatories (NSAIDs) for 6 h prior to visit. Asthmatics were asked to stop their asthma medication if safe to do so: no cromoglycate, nedocromil, short-acting beta-agonists or ipratroprium bromide for 6 h prior to the visit, no long-acting beta-agonists for 12 h prior to the visit and no theophyllines for 24 h prior to the visit.

### Data collection

Information was collected using standardized methods including questionnaires, lung function and atopy testing, blood collection and sputum induction.

### Questionnaire

This was largely based on the ISAAC Phase II, with additional questions on asthma control.^24^ The standard ISAAC definition of chronic severe asthma was used: current wheeze with more than four attacks of wheezing in the past 12 months, or more than one night per week sleep disturbance, or wheeze affecting speech.^25^ In addition, a stricter definition of severe asthma (>12 attacks of wheezing in the past 12 months) was used (aligned more closely with Global Initiative for Asthma (GINA) 2008 guidelines).^26^

### Skin prick tests

Skin prick tests (SPT) were carried out as described previously,^27^^,^^28^ with atopy defined as the presence of one or more weal of ≥3 mm in response to at least one of a panel of eight or more commercially available allergens (ALK, Stallergenes, Greer, Immunotech), including house dust mite (*Dermatophagoides pteronyssinus*), tree pollen mix, grass pollen mix, cat and dog dander, *Alternaria tenuis*, *Penicillium* mix, plus locally relevant allergens^10^ (e.g. *Blomia tropicalis* (dust mite), German cockroach, American cockroach, A*spergillus fumigatus* and *Cladosporium*). Histamine and saline were used as positive and negative controls, respectively.

### Lung function testing

Lung function testing was conducted according to American Thoracic Society (ATS) criteria. Global Lung Function Initiative (GLI-2012) reference values were used to calculate z-scores, taking into account age, sex, height and ethnicity.^29^

### Fractional exhaled nitric oxide (FeNO)

FeNO was an optional part of the protocol and was measured in three centres using the Bedfont NOBreath device (Bedfont Scientific Ltd, Maidstone, UK) (Ecuador and Uganda) and Hypair FeNO analyser (Medisoft, Sorinnes, Belgium) (New Zealand). The two instruments were compared in a subgroup of participants in New Zealand and no substantial differences were observed (data not shown). Two or three measurements were taken from each participant and the mean value calculated. FeNO was considered to be elevated if the mean value was >35 parts per billion (ppb) for participants aged <12 years or >50ppb for participants aged ≥12 years.

### Blood eosinophils

In each centre, 5 ml of peripheral blood was collected into an EDTA tube and processed within 4 h at the local laboratory. A full blood count was conducted according to standard procedures and included an eosinophil count.

### Sputum induction

Sputum induction was conducted using a standardized protocol that we have used previously,^30^ adapted from Gibson *et al*.^31^ Aerosolized hypertonic saline (4.5% w/v) was produced using an ultrasonic nebulizer (DeVilbiss Ultraneb 3000, Langen, Germany) and administered orally through a mouthpiece (Hans-Rudolph Inc., Kansas City, USA) for increasing intervals from 30 s to 4 min, to a total of 15.5 min. Spirometry was conducted between intervals to monitor forced expiratory volume in one s (FEV_1_), and salbutamol was administered if FEV_1_ dropped to 75% of predicted or less. Participants were subsequently encouraged to produce sputum in a sterile plastic container. In the UK centre, 5% hypertonic saline was used because 4.5% could not be sourced, and in the Ecuador, Uganda and UK centres, disposable tubing was used for infection control reasons or because suitable disinfection facilities were not available on site. Sputum was processed according to a well‐characterized protocol,^32^ and the resulting cell suspension used to prepare cytospin slides stained using a Diff-Quik^®^ fixative and stain set (Dade Behring, Deerfield, IL). Sputum slides were read in Wellington, New Zealand, with the exception of the slides produced in Brazil (which could not be shipped overseas due to ethical restrictions): these were therefore read in Brazil, with a sample of slides being remotely checked (using microscopy images) by the group in Wellington. Asthma inflammatory phenotypes were defined as: eosinophilic: ≥2.5% eosinophils and <61% neutrophils; mixed granulocytic: ≥2.5% eosinophils and ≥61% neutrophils; neutrophilic: <2.5% eosinophils and ≥61% neutrophils; and paucigranulocytic: <2.5% eosinophils and <61% neutrophils.^30^^,^^32^ Analyses were repeated using a 1% cut-off for eosinophils^11^ or using a 54% cut-off for neutrophils (as done in other paediatric studies).^33^^,^^34^ Results are also presented excluding low-quality slides (<400 total non-squamous cells, and >30% squamous cells). Induced sputum testing was repeated after approximately 3 months in about 50 asthmatics per centre.

### Data analysis

Data were analysed using Stata 16. The focus was on defining categories within the groups of asthmatics; however in each centre, a comparison was also made with non-asthmatics. Initial descriptive analyses involved simple means and percentages; 95% confidence intervals (95% CI) were calculated for key means and percentages, and population attributable risks (PARs)^4^ and 95% CIs where appropriate. To enable valid comparisons between centres, logistic regression and multinomial regression analyses were also conducted, adjusting for age and sex. The post-estimation margins command was used to calculate predicted proportions with eosinophilic asthma (eosinophilic + mixed granulocytic) at age 15 years by sex and by centre. Differences between inflammatory phenotype groups were tested with chi squared tests or t tests/analysis of variance (ANOVA). Logistic regression and linear regression were used to calculate estimates for associations between EA/NEA and clinical characteristics, adjusted for centre, age and sex.

## Results

Table 1 summarizes the participant characteristics (study centre characteristics have been described previously^10^). Overall, 998 asthma cases and 356 controls were recruited (a detailed breakdown of recruitment per centre is provided in Supplementary Materials, available as Supplementary data at *IJE* online). All centres included children and adolescents (age range 8–20 years), except for the UK centre for which the participants were 26–27 years. Asthma was more often diagnosed by a doctor in NZ and the UK than in Brazil, Ecuador and Uganda, and the proportion with severe asthma was higher in Brazil and Uganda compared with the other centres. Over half of asthma cases in Ecuador and just over a quarter of cases in Brazil and Uganda had no asthma medication in the previous 12 months. Use of inhaled corticosteroids was most common in NZ and the UK and rare in Ecuador, and systemic corticosteroids were often used in Uganda (Table 1). Table 2 shows the clinical characteristics of asthma cases and controls by centre. The proportion of skin prick test (SPT)-positive cases ranged from 35% (Ecuador) to 84% (Brazil). The proportion of controls who were SPT+ ranged from 13% (Uganda) to 65% (Brazil). We also calculated the PARs of SPT positivity for asthma (not shown in table): Brazil (50%), Ecuador (25%), Uganda (43%), New Zealand (67%) and the UK (59%), with an overall estimate of 48% (95% CI 44%–52%). The proportion of participants who produced a sample from the sputum induction procedure ranged from 74% (181, Brazil) to 93% (229, UK) (Table 3). Of these, the proportion of countable sputum slides ranged from 48% (111, UK) to 95% (332, New Zealand). Other than the differences between centres in the proportion of participants with sputum phenotype available, there were few substantial differences in the characteristics of participants with and without sputum phenotype available (Supplementary Table S1, available as Supplementary data at *IJE* online). The prevalence of airway eosinophilia (either eosinophilic or mixed granulocytic) was 50% in New Zealand and about one-third (32–35%) in the UK and LMIC centres. Remaining cases were predominantly paucigranulocytic, with little evidence of neutrophilic asthma (<10% prevalence) across four centres. The exception was Uganda, where over a third (35%, *n* = 34) of all cases had the neutrophilic phenotype. However, the proportion with a neutrophilic pattern was even higher in the controls (60%, *n* = 12).

**Table 2:** Clinical characteristics of asthma cases and controls by centre

Centre	Brazil (Salvador)	Ecuador (Quininde)	Uganda (Entebbe)	New Zealand (Wellington)	UK (Bristol)
**Cases**	*n *= 204	*n* = 176	*n* = 207	*n* = 235	*n* = 176
Lung function^a^mean (SD), range	*n* = 198	*n* = 172	*n* = 138	*n* = 234	*n *= 170
FEV_1_	2.89 (0.63), 1.44–4.84	2.10 (0.46), 1.16–3.79	2.60 (0.48), 1.68–4.06	2.76 (0.94), 1.13–5.37	3.49 (0.78), 1.62–5.61
FEV_1_ z-score	−0.98 (1.02), −4.03–1.36	−0.30 (0.96), −3.03–2.17	−0.21 (0.89), −2.78–2.23	−0.49 (1.07), −3.52–2.36	−0.40 (1.15), −4.12–3.59
FVC	3.51 (0.86), 0.42–6.80	2.34 (0.52), 1.28–4.23	3.05 (0.70), 1.77–8.01	3.39 (1.13), 1.36–6.05	4.28 (1.05), 1.80–7.78
FVC z-score	−0.57 (1.23), −7.51–2.37	−0.49 (1.01), −3.30–2.71	0.10 (1.15), −1.94–7.14	0.09 (0.98), −2.56–2.97	−0.11 (1.06), -4.52–3.74
FEV_1_/FVC	0.83 (0.10), 0.40–1.00	0.90 (0.06), 0.74–1.00	0.86 (0.09), 0.49–1.00	0.82 (0.07), 0.59–0.99	0.82 (0.08), 0.47–0.97
FEV_1_/FVC z-score	−0.72 (1.45), −4.67–2.76	0.37 (1.04), −2.34–2.61	−0.41 (1.29), −4.25–2.51	−0.82 (1.05), −3.62–2.29	−0.45 (1.05), −4.05–2.20
Skin prick test positivity	168 (84%)	62 (35%)	103 (53%)	187 (80%)	124 (82%)
Blood eosinophils	*n* = 196	*n *= 176	*n* = 196	*n* = 183	*n* = 125
absolute values (10^9^/L) median (IQR), (range)	0.39 (0.23–0.56), (0–1.30)	0.55 (0.29–0.81), (0–3.4)	0.24 (0.12–0.40), (0–3.00)	0.4 (0.2–0.7), (0–1.9)	0.20 (0.10–0.38), (0.02–0.83)
**Controls**	*n* = 40	*n* = 67	*n* = 50	*n* = 132	*n* = 67
Lung function^a^	*n *= 39	*n* = 67	*n *= 36	*n *= 131	*n* = 65
mean (SD), range					
FEV_1_	3.32 (0.72), 2.32–5.17	2.10 (0.37), 1.35–3.19	2.68 (0.65), 1.57–4.17	3.03 (0.98), 1.31–5.42	3.85 (0.90), 2.12–6.32
FEV_1_ z-score	−0.35 (0.95), −2.47–1.99	0.12 (1.06), −1.99–2.80	0.03 (1.24), −1.97–4.58	−0.11 (0.98), −2.41–2.60	−0.12 (1.09), −2.62–2.76
FVC	3.69 (0.84), 2.74–6.31	2.29 (0.40), 1.53–3.46	3.08 (0.77), 1.65–5.06	3.51 (1.15), 1.44–6.31	4.60 (1.22), 2.40–8.11
FVC z-score	−0.58 (1.17), −3.14–2.43	−0.27 (1.11), −2.66–2.07	0.17 (1.37), −2.34–4.13	−0.04 (0.90), −2.16–2.28	−0.10 (1.09), −2.73–3.12
FEV_1_/FVC	0.90 (0.06), 0.73–1.00	0.92 (0.05), 0.81–1.00	0.88 (0.10), 0.48–1.00	0.87 (0.05), 0.67–0.99	0.85 (0.06), 0.70–0.97
FEV_1_/FVC z-score	0.36 (1.10), −2.40–2.36	0.80 (1.05), −1.20–2.95	−0.02 (1.37), −3.92–1.93	−0.07 (0.88), −2.68–2.14	−0.05 (0.91), −2.11–2.56
Skin prick test positivity	26 (65%)	9 (14%)	6 (13%)	51 (40%)	19 (29%)
Blood eosinophils	*n = 39*	*n = 67*	*n = 47*	*n = 109*	*n = 56*
absolute values (10^9^/L) median (IQR), (range)	0.19 (0.08–0.27), (0–2.71)	0.42 (0.23–0.88), (0–1.88)	0.15 (0.09–0.32), (0.03–0.83)	0.2 (0.1–0.3), (0–2.2)	0.10 (0.06–0.16), (0.02–0.51)

a Absolute values (L) and GLI-2012 z-scores.

SD, standard deviation; FEV_1_, forced expiratory volume in 1 s; FVC, forced vital capacity; IQR, interquartile range; GLI, Global Lung Function Initiative.

**Table 3:** Sputum slide results by centre

Centre	Brazil	Ecuador	Uganda	New Zealand	UK	Total
Number (%) of participants who provided sputum sample	181 (74%)	244 (91%)	221 (86%)	350 (88%)	229 (93%)	1225 (87%)
Number of participants with countable sputum slide(s) (% of those who provided sample)	137 (76%)	183 (75%)	118 (53%)	332 (95%)	111 (48%)	881 (72%)
**Asthma cases, *n***	115	125	98	207	78	623
Sputum inflammatory phenotype:						
Eosinophilic	38 (33%)	35 (28%)	25 (25%)	99 (48%)	24 (31%)	221 (35%)
Mixed granulocytic	2 (2%)	5 (4%)	8 (8%)	5 (2%)	2 (3%)	22 (4%)
Neutrophilic	5 (4%)	8 (6%)	34 (35%)	14 (7%)	6 (8%)	67 (11%)
Paucigranulocytic	70 (61%)	77 (62%)	31 (32%)	89 (43%)	46 (59%)	313 (50%)
Repeat sputum slide, *n*	40	36	12	107	11	206
Same phenotype (EA or NEA)	27 (68%)	25 (69%)	9 (75%)	72 (67%)	6 (55%)	139 (67%)
Changed: EA to NEA	6	4	0	18	4	32 (15%)
Changed: NEA to EA	7	7	3	17	1	35 (17%)
**Controls, *n***	20	41	20	104	31	216
Sputum inflammatory phenotype:						
Eosinophilic	4 (20%)	3 (7%)	2 (10%)	11 (11%)	3 (10%)	23 (11%)
Mixed granulocytic	0	0	1 (5%)	1 (1%)	0	2 (1%)
Neutrophilic	4 (20%)	1 (2%)	12 (60%)	11 (11%)	3 (10%)	31 (14%)
Paucigranulocytic	12 (60%)	37 (90%)	5 (25%)	81 (78%)	25 (81%)	160 (74%)

EA, eosinophilic asthma (eosinophilic or mixed granulocytic); NEA, non-eosinophilic asthma (neutrophilic or paucigranulocytic).

**Figure 1:**
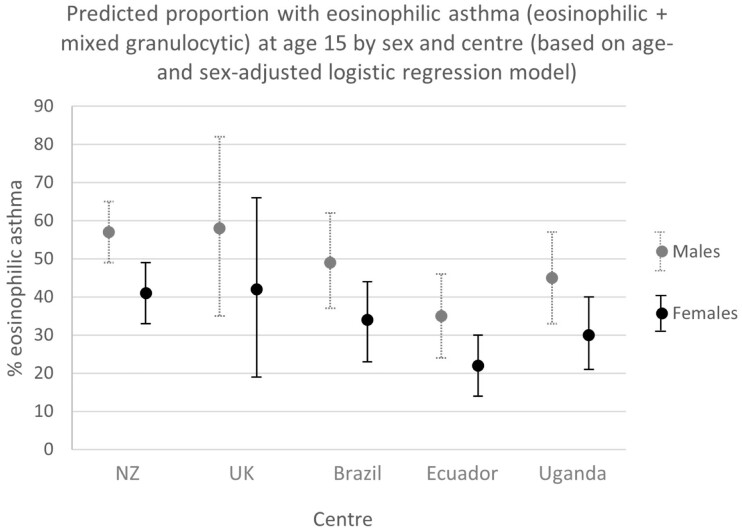
Predicted proportion with eosinophilic asthma (eosinophilic + mixed granulocytic) at age 15 by sex and centre, from age- and sex-adjusted logistic regression model, *n *= 623

There was a weak association between increasing age and reduced odds of EA (eosinophilic + mixed granulocytic) compared with NEA (neutrophilic + paucigranulocytic), and a strong association between sex and phenotype, with males more likely to be EA than females. The age-and sex-adjusted odds ratios (Table 4) indicate that the UK centre had a similar odds of EA to New Zealand (adjusted OR 1.04, 95% CI 0.37–2.94). The odds of EA in Ecuador and Uganda remained lower than in New Zealand but the difference between Brazil and New Zealand was attenuated (Table 4). Figure 1 shows the predicted proportion with EA at age 15 by sex and centre, based on the regression model used for Table 4. Females had a consistently lower proportion of EA than males across centres, although the confidence intervals overlapped in all centres except New Zealand. The predicted proportions with EA at ages 20 and 25 showed a similar pattern.

**Table 4:** Association between centre and EA in asthma cases using logistic regression, *n *= 623

Centre	EA (eosinophilic + mixed)	NEA (neutrophilic + paucigranulocytic)	Unadjusted odds ratio (OR) (95% CI) for EA vs NEA	Adjusted for age OR (95% CI)	Adjusted for age and sex OR (95% CI)
NZ (baseline)	104 (50%)	103	1.0	1.0	1.0
UK	26 (33%)	52	0.50 (0.29–0.85)	1.04 (0.37–2.89)	1.04 (0.37–2.94)
Brazil	40 (35%)	75	0.53 (0.33–0.85)	0.68 (0.39–1.17)	0.73 (0.42–1.27)
Ecuador	40 (32%)	85	0.47 (0.29–0.74)	0.41 (0.25–0.67)	0.40 (0.24–0.66)
Uganda	33 (34%)	65	0.50 (0.31–0.83)	0.54 (0.33–0.90)	0.62 (0.37–1.04)
Age (years)				0.94 (0.87–1.01)	0.95 (0.88–1.02)
Sex (female)					0.53 (0.37–0.74)

EA, eosinophilic asthma; NEA, non-eosinophilic asthma.

A multinomial model (comparing EA with paucigranulocytic and neutrophilic with paucigranulocytic) confirmed the higher proportion of participants with the neutrophilic phenotype in Uganda compared with New Zealand when adjusting for age and sex (Supplementary Table S2, available as Supplementary data at *IJE* online). This model also suggested a higher proportion of neutrophilic phenotype in the UK centre compared with New Zealand when adjusted for age and sex, but with a very wide confidence interval.

When we assessed inflammatory phenotype stability in a subgroup of asthmatics over an approximately 3-month period, we found that 139 of 206, 67% (95% CI 61%–74%), had the same general phenotype (EA or NEA) across the two tests, ranging from 55% (6) in the UK to 75% (9) in Uganda, with roughly equal numbers switching from EA to NEA and vice versa (Table 3). The characteristics of the inflammatory phenotypes for all centres combined are summarized in Table 5 (details for each centre are provided in Supplementary Table S3, available as Supplementary data at *IJE* online). Severe asthma was more common in EA (eosinophilic and mixed) than in NEA (neutrophilic and paucigranulocytic) (chi square = 15.5, *P *<0.001). A higher proportion of NEA had well-controlled asthma in the past week compared with EA. The mean FEV_1_ scores were lower in the EA groups than in the NEA groups (t = -3.90, *P *=* *0.0001). Blood eosinophil levels varied by inflammatory phenotype [Kruskal–Wallis (chi square = 85.51, *P *=* *0.0001)]: highest in the eosinophilic group and the lowest in the neutrophilic group. FeNO was also more often elevated in the EA groups than in the NEA groups (chi square = 84.69, *P *<0.001) and the proportion with a positive skin prick test was highest in the eosinophilic group. These differences remained when adjusting for centre, age and sex (Table 5). We also calculated the PARs of EA (eosinophilic or mixed granulocytic) for asthma (not shown in table): these were Brazil (18%), Ecuador (27%), New Zealand (44%), Uganda (22%) and the UK (18%), with an overall estimate of 30% (95% CI 25%–33%).

**Table 5:** Clinical characteristics by inflammatory phenotype for all centres combined

Inflammatory phenotype	Eosinophilic	Mixed granulocytic	Neutrophilic	Paucigranulocytic	Odds ratio (or coefficient) (95% CI) for characteristic comparing EA with NEA, adjusted for centre, age and sex
	
	EA		NEA		
Clinical characteristic, *n* (%)	*n *= 221	*n *= 22	*n* = 67	*n* = 313	
Severe asthma^a^ (ISAAC)	132 (60%)	15 (68%)	29 (43%)	135 (45%)	2.09 (1.48–2.97)
Severe asthma (>12 attacks^a^)	36 (16%)	3 (14%)	8 (12%)	21 (7%)	2.31 (1.35–3.97)
Well-controlled asthma in past week (ACQ score <1.5)	160 (77%)	14 (64%)	54 (82%)	249 (82%)	0.56 (0.36–0.88)
ICS (preventer inhaler)^a^	102 (46%)	10 (45%)	16 (24%)	103 (33%)	1.49 (0.98–2.29)
Skin prick test positive	180 (84%)	13 (59%)	38 (57%)	174 (57%)	3.22 (2.09–4.96)
Blood eosinophils absolute values (10^9^/L) median (range)	0.55 (0–2.99)	0.33 (0.06–1.18)	0.22 (0.01–2.95)	0.29 (0–3.40)	coefficient 0.21 (0.14–0.28)
FEV_1_ z-score mean (SD)	−0.64 (0.95)	−0.68 (1.46)	−0.31 (0.89)	−0.32 (1.02)	coefficient −0.33 (−0.49 – −0.16)
Elevated FeNO level (3 centres)	108 (69%)	11 (61%)	18 (32%)	42 (22%)	6.39 (4.09–9.99)

EA, eosinophilic asthma; NEA, non-eosinophilic asthma; SD, standard deviation; ISAAC, International Study of Asthma and Allergies in Childhood; ACQ; Asthma Control Questionnaire; ICS, inhaled corticosteroids; FEV_1_, forced expiratory volume in 1 s; FeNO, fractional exhaled nitric oxide.

a In the past 12 months.

Supplementary Table S4 (available as Supplementary data at *IJE* online) shows the sputum results excluding low-quality slides (<400 total non-squamous cells and >30% squamous cells). Findings changed little; for example, the proportions with EA (eosinophilic + mixed) changed from 34% to 32% in the UK, 50% to 52% in New Zealand, 35% to 32% in Brazil, 32% to 33% in Ecuador and 33% to 29% in Uganda. Using a 1% eosinophil cut-off, the proportions with EA ranged from 40% (Ecuador) to 69% (New Zealand) (Supplementary Table S5, available as Supplementary data at *IJE* online). Similarly, changing the neutrophil cut-off to 54% did not considerably alter the findings; the largest changes in the neutrophilic phenotype proportion were 35% to 40% in Uganda and 8% to 14% in the UK (Supplementary Table S5). Table 6 shows the sputum results separately for participants who regularly used inhaled corticosteroids (ICS) in the past 12 months (as ICS may reduce airway eosinophilia),^3^ and for those who did not. The proportions with EA were lower among those who did not regularly use ICS compared with regular ICS users.

**Table 6:** Sputum slide results by centre, in asthma cases with regular inhaled corticosteroid (ICS) use in the past 12 months (ICS users) and non-ICS users

	Brazil regular ICS users	Brazil not regular ICS users	Ecuador ICS users^a^	Ecuador not ICS users	Uganda ICS users^a^	Uganda OCS users (with no ICS use)	Uganda not ICS users	NZ regular ICS users	NZ not regular ICS users	UK regular ICS users	UK not regular ICS users
*n*	11	104	6	119	11	30	87	82	125	38	40
Sputum inflammatory phenotype:											
Eosinophilic	6 (55%)	32 (31%)	0 (0%)	35 (29%)	4 (36%)	8 (27%)	21 (24%)	44 (54%)	55 (44%)	13 (34%)	11 (28%)
Mixed granulocytic	0 (0%)	2 (2%)	0 (0%)	5 (4%)	2 (18%)	4 (13%)	6 (7%)	3 (4%)	2 (2%)	2 (5%)	0 (0%)
Neutrophilic	0 (0%)	5 (5%)	0 (0%)	8 (7%)	2 (18%)	12 (40%)	32 (37%)	7 (8%)	7 (6%)	2 (5%)	4 (10%)
Paucigranulocytic	5 (45%)	65 (62%)	6 (100%)	71 (60%)	3 (27%)	6 (20%)	28 (32%)	28 (34%)	61 (49%)	21 (56%)	25 (62%)
Repeat sputum slide											
Same phenotype (EA or NEA)	1 (50%)	26 (68%)	0 (0%)	25 (74%)	1 (100%)	4 (80%)	8 (73%)	24 (63%)	48 (70%)	4 (50%)	2 (67%)
Changed:											
EA to NEA	1	5	0	4	0	0	0	6	12	3	1
NEA to EA	0	7	2	5	0	1	3	8	9	1	0

OCS, oral corticosteroids; EA, eosinophilic asthma (eosinophilic or mixed); NEA, non-eosinophilic asthma (neutrophilic or paucigranulocytic); ICS, inhaled corticosteroids.

a No regular ICS use, only when wheezy.

## Discussion

This is the first time that sputum induction has been used in a standardized manner to compare asthma inflammatory phenotypes in centres in high-income countries (HICs) and low-and-middle-income countries (LMICs). The proportion of EA was lower in the LMICs than in the HICs, and the majority of asthma cases in all centres were non-eosinophilic. With the exception of Uganda, the paucigranulocytic phenotype, characterized by an absence of detectable airway inflammation, made up the majority of NEA. There are several key findings that should be considered.

First, for the four centres that involved children and adolescents, this study confirms previous research in HICs that only about one-half of asthmatics have EA, and it shows for the first time that only about one-third of asthmatics in the centres in LMICs have EA. This adds to previous evidence that a substantial proportion (more than half) of asthma involves non-eosinophilic inflammatory phenotypes, and shows, for the first time to our knowledge, that this is the case in LMICs as well as HICs. Although it is possible that some NEA cases may represent EA in which inhaled corticosteroid (ICS) treatment has suppressed airway eosinophilia,^3^ it is unlikely that this accounts for the majority of NEA cases^11^ since most have persistent symptoms, and in most LMICs less than 20% of asthma cases regularly used ICS. Moreover, Table 6 shows that the proportions with EA were actually lower among those not regularly using ICS than among those using ICS, indicating that ICS use has not biased the phenotype distribution towards NEA.

The crude proportions of asthma cases who were classified as EA (eosinophilic + mixed) were 32–35% in the LMICs (Brazil, Ecuador, Uganda); among the HICs it was 50% in New Zealand and 34% in the UK. However, after adjusting for age and sex, the estimate for the UK was comparable to New Zealand (the UK participants were older and the odds of EA decreased with age). The lower proportions of EA in Uganda and Ecuador compared with New Zealand remained after adjustment for age and sex, but the difference between Brazil and New Zealand attenuated. However, the comparisons of the UK centre with the other four centres should be treated with caution since there was no overlap in age range between the UK centre and the other centres. There was a strong association between sex and inflammatory phenotype, with a lower proportion of EA among females consistently across centres. Asthma prevalence overall is higher in males among children and becomes higher in females in adolescence, with the switch coinciding with puberty onset.^35^ However, to our knowledge, there are no previous reports of sex differences in asthma phenotypes and this finding warrants further investigation.

Second, there was a high prevalence of sputum neutrophilia in Uganda. This may be in part due to the low proportion with eosinophilia, but interestingly, the proportion was actually lower in the asthma cases (35%) than in the controls (60%). Furthermore, in all centres (with the exception of Ecuador) there was a higher proportion of neutrophilia in the controls (14% overall) than in the cases (11%). Previous studies have found neutrophilia to be associated with more severe asthma,^11^ but this was not observed in the Uganda cases in the current study (Supplementary Table S3). It is possible that the high proportion with neutrophilia reflects background (i.e. non-asthmatic) neutrophilic inflammation due to environmental exposures (e.g. indoor air pollution, increased risk of infections, exposure to animals, endotoxin exposure).

Third, the most striking finding is the high proportion of cases with no granulocytic inflammation (i.e. paucigranulocytic) in all centres. This supports findings from previous studies in HICs,^30^ which have shown that a high proportion of asthmatics appear to have no clear evidence of airways inflammation, thus raising the possibility that non-inflammatory mechanisms (e.g. neural mechanisms^36^) may be involved. Alternatively, it is possible that the inflammatory phenotype is unstable, and that children with EA may only show intermittent eosinophilic inflammation, particularly during exacerbation.^30^ This is possible because in the current study clinic visits were delayed by at least 4 weeks in cases of an acute exacerbation of asthma, in order to ensure sputum induction safety and comparability. However, some previous studies have shown phenotypes to be relatively stable,^11^^,^^37^^,^^38^ which corresponds to our findings in a subset of asthmatics: 67% had the same phenotype (EA or NEA) in the repeat sputum assessments. The requirement to delay the study visit in the event of a recent exacerbation or respiratory tract infection may also explain the apparent discrepancy of the proportion with severe asthma in the past year and the proportion with well-controlled asthma in the past week. It is also possible that those with the paucigranulocytic phenotype have been misidentified as asthma cases, or may have mild/intermittent asthma; alternatively, it could represent low-level eosinophilic inflammation occurring outside the central airways.^39^

We endeavoured to obtain random population samples of asthmatics in each centre, by taking random samples in schools and by using existing cohort studies. The participants were chosen to be a representative sample of asthmatics in general rather than focusing on severe asthma, as is reflected by the clinical indicators. We used a consistent definition across centres and inflammatory phenotypes, so it is noteworthy that we found only small differences in chronic asthma severity between eosinophilic (60%), mixed granulocytic (68%), neutrophilic (43%) and paucigranulocytic (43%) asthma. These proportions are relatively high, given that most participants had well-controlled asthma, but this reflects the ISAAC definition that is based on symptoms in the past year^25^ and yields higher estimates of chronic asthma severity than do other definitions that focus on acute clinical severity. Moreover, previous studies have shown that the asthma definition used here is strongly associated with clinical diagnosis and objective measures of asthma.^22^ In addition, given that the proportion of asthma cases previously diagnosed by a physician is much lower in LMICs (as displayed in Table 1, this ranges from 61% to 74%), the definition we used based on the ISAAC questionnaire is preferable.

Although median blood eosinophil levels were highest in the eosinophilic group and lowest in the neutrophilic group, there was a wide range of levels within each inflammatory phenotype group, such that some participants in the NEA groups would have been classified as eosinophilic based on their blood results. Although blood eosinophil results are often used in clinical practice as they are easier to measure, the sputum counts provide a more specific characterization of asthma inflammatory phenotypes as they capture the level of inflammation in the airways, rather than systemic inflammation which may be due to several causes other than asthma.

Some limitations of this study should also be acknowledged. Standardizing data collection (particularly the sputum induction) was difficult, and in some centres it was difficult to obtain readable slides from sputum samples from a high proportion of participants. In particular, only 48% of the slides from Bristol were readable (Table 3), often due to squamous cell contamination. The reasons for this are unclear. However, other than differences by centre, there were few substantial differences in the characteristics of participants with and without sputum phenotype available (Supplementary Table S1), so our results are unlikely to be affected by selection bias. Also, findings did not change markedly when we restricted our analyses to high-quality slides and this appears unlikely to have introduced any phenotype bias (Supplementary Table S4).

The prevalence of atopy among participants in Brazil was somewhat higher than expected and it is possible that those with atopy were over-represented in the sample for this study, which may have led to an overestimate of the proportion with EA from the Brazil centre. The population atrributable risks of skin prick test positivity for asthma were higher than expected, which could also be due to over-representation of atopic participants in some centres.

## Conclusion

In conclusion, this study confirms that most asthma is non-eosinophilic (often paucigranulocytic, with no detectable sign of airways inflammation) across varied geographical and socioeconomic environments. After adjustment for age and sex, higher proportions of EA were estimated for the HICs (New Zealand and the UK) compared with the middle-income country (Brazil) and the low-income countries (Uganda and Ecuador), with a suggestive trend. In addition, a strong association was observed between sex and phenotype, with males more likely to be EA than females. These findings potentially have major implications for asthma prevention and management globally. They also highlight the urgent need to conduct further research elucidating the environmental exposures and triggers in NEA, and determining the underlying aetiology in these cases. In particular, there is a need to develop new therapies, management and intervention strategies which specifically target and improve clinical outcomes in NEA,^1^ as currently a large proportion of asthmatics are treated with drugs that are likely to be ineffective in many cases.

## The WASP Study group

UK, London: Neil Pearce,* Lucy Pembrey,* Steven Robertson, Karin van Veldhoven,* Charlotte E Rutter,* Sinead Langan, Sarah Thorne, Donna Davoren. UK, Bristol: John Henderson, Susan Ring, Elizabeth Brierley, Sophie Fitzgibbon, Simon Scoltock, Amanda Hill. Brazil, Leading Group: Alvaro Cruz,* Camila Figueiredo,* Mauricio Barreto.* Brazil, ProAR collaborators/associates: Cinthia Vila Nova Santana, Gabriela Pimentel, Gilvaneide Lima, Valmar Bião Lima, Jamille Fernandes. Brazil, laboratory students/associates: Tamires Cana Brasil Carneiro, Candace Andrade, Gerson Queiroz, Anaque Pires, Milca Silva, Jéssica Cerqueira. Ecuador: Philip Cooper,* Martha Chico, Cristina Ardura-Garcia, Araceli Falcones, Aida Y Oviedo, Andrea Zambrano. New Zealand: Jeroen Douwes,* Collin Brooks,* Hajar Ali, Jeroen Burmanje. Uganda: Harriet Mpairwe,* Irene Nambuya, Pius Tumwesige, Milly Namutebi, Marble Nnaluwooza, Mike Mukasa.

*Writing Group for this paper.

## Ethics approval

Ethical approval was obtained from the London School of Hygiene & Tropical Medicine (LSHTM) ethics committee (ref: 9776) and the participating study centres (see Supplementary Materials). Informed consent was obtained from all participants or their parents/carers.

## Supplementary Material

dyac173_Supplementary_Data

## Data Availability

The data underlying this article will be shared on reasonable request to the corresponding author.
