# Supplementary material for: Asthma inflammatory phenotypes on four continents: most asthma is non-eosinophilic
Source: Int J Epidemiol. 2022 Aug 30;52(2):611–23. doi: 10.1093/ije/dyac173 (PMC10114118; doi:10.1093/ije/dyac173)
Supplement: dyac173_Supplementary_Data [file dyac173_supplementary_data.docx]

**SUPPLEMENTARY MATERIALS**

**Ethics approval**

Ethics approval for the study has been obtained from the LSHTM ethics committee (ref: 9776) and in all five study centres. Informed consent was obtained from all participants or their parents/carers before taking part.

ALSPAC: Ethical approval for the UK-arm of the study was obtained from the ALSPAC Ethics and Law Committee, and the Local Research Ethics Committees. Consent for biological samples has been collected in accordance with the Human Tissue Act (2004). REDcap was used to collect the ALSPAC data (<http://projectredcap.org/resources/citations/>). Informed consent for the use of data collected via questionnaires and clinics was obtained from participants following the recommendations of the ALSPAC Ethics and Law Committee at the time. The ALSPAC study website contains details of all the data that is available through a fully searchable data dictionary and variable search tool (<http://www.bristol.ac.uk/alspac/researchers/our-data/)/>.

**Recruitment per centre**

In Brazil, 685 individuals were contacted; 298 (44%) agreed to a clinic visit, of whom 250 attended. Participants were recruited from the SCAALA cohort (14 asthmatics and 15 non-asthmatics) or schools (190 asthmatics and 25 non-asthmatics). In Ecuador, participants were recruited from a cohort study (89 asthmatics and 67 non-asthmatics) or schools (87 asthmatics); the response rate was over 95%. In New Zealand, participants were recruited from either a birth cohort (from which 908 individuals were invited and 386 (43%) agreed and completed the questionnaire) or community recruitment (contact details for 1279 individuals were obtained, of whom 824 (64%) confirmed that they wanted to participate (n=455 refused or were ineligible/uncontactable after initial contact)). For this study, 134 participants were recruited from the birth cohort (57 asthmatics and 77 non-asthmatics) and 233 from the community (178 asthmatics and 55 non-asthmatics). In Uganda, recruitment was through schools; of 6353 parents invited, 1750 (28%) attended a parents’ meeting about the study. Of those attending the meeting, 1734 (99%) consented to their child taking part. 207 asthmatics and 50 non-asthmatics were selected for this study. In the UK, 963 ALSPAC participants were invited; 54 (6%) declined and 390 (40%) accepted; following screening for eligibility, clinic visits were completed for 243.

**Table S1.** Comparison of participants with phenotype and those without phenotype available.

|  | Sputum phenotype available | Sputum phenotype not available | ꭓ^2^, p  t, p  z, p |
| --- | --- | --- | --- |
| Asthma cases | n=623 | n=375 |  |
| **Centre,** n (%) |  |  |  |
| NZ | 207 (88%) | 28 | 119.4, <0.001 |
| UK | 78 (44%) | 98 |  |
| Brazil | 115 (56%) | 89 |  |
| Ecuador | 125 (71%) | 51 |  |
| Uganda | 98 (47%) | 109 |  |
| **Age** (mean, range) |  |  |  |
| NZ | 14.3 (8.6 – 20.3) | 12.6 (9.0 – 17.9) | t=-2.80, 0.006 |
| UK | 25.9 (24.9 – 27.2) | 25.9 (24.6 – 27.3) | t=0.18, p=0.86 |
| Brazil | 18.2 (12.5 – 23.8) | 18.4 (12.0 – 23.9) | t=0.69, 0.49 |
| Ecuador | 12.2 (10.3 – 16.9) | 11.8 (10.3 – 14.3) | t=-1.52, 0.13 |
| Uganda | 15.4 (12.0 – 17.9) | 15.7 (12.2 – 18.0) | t=1.10, 0.27 |
| **Sex** n (%) female |  |  |  |
| NZ | 103 (50%) | 10 (36%) | 1.95, 0.16 |
| UK | 58 (74%) | 73 (74%) | 0.0004, 0.98 |
| Brazil | 81 (70%) | 53 (60%) | 2.64, 0.10 |
| Ecuador | 59 (47%) | 17 (33%) | 2.84, 0.09 |
| Uganda | 73 (74%) | 82 (75%) | 0.02, 0.90 |
| **Severe asthma* (ISAAC)**  n (%) |  |  |  |
| NZ | 98 (47%) | 14 (50%) | 0.07, 0.79 |
| UK | 35 (45%) | 44 (45%) | 0.000, 0.997 |
| Brazil | 71 (70%) | 47 (59%) | 2.29, 0.13 |
| Ecuador | 52 (42%) | 27 (53%) | 1.77, 0.18 |
| Uganda | 55 (56%) | 81 (74%) | 7.58, 0.006 |
| **Severe asthma (>12 attacks*)**  n (%) |  |  |  |
| NZ | 29 (14%) | 7 (25%) | 2.30, 0.13 |
| UK | 13 (17%) | 14 (14%) | 0.19, 0.66 |
| Brazil | 9 (8%) | 8 (9%) | 0.09, 0.77 |
| Ecuador | 0 (0%) | 0 (0%) |  |
| Uganda | 17 (17%) | 22 (20%) | 0.27, 0.60 |
| **Well controlled asthma in past week (ACQ score<1.5)**  n (%) |  |  |  |
| NZ | 149 (80%) | 21 (88%) | 0.75, 0.39 |
| UK | 66 (87%) | 88 (93%) | 1.58, 0.21 |
| Brazil | 76 (67%) | 68 (78%) | 3.21, 0.07 |
| Ecuador | 120 (96%) | 48 (94%) | 0.30, 0.59 |
| Uganda | 66 (69%) | 53 (74%) | 0.47, 0.49 |
| **ICS (preventer inhaler) ***  n (%) |  |  |  |
| NZ | 142 (69%) | 20 (71%) | 0.09, 0.76 |
| UK | 50 (72%) | 44 (59%) | 3.02, 0.08 |
| Brazil | 22 (20%) | 21 (24%) | 0.60, 0.44 |
| Ecuador | 6 (8%) | 0 | 2.26, 0.13 |
| Uganda | 11 (12%) | 22 (21%) | 3.07, 0.08 |
| **Skin prick test positive**  n (%) |  |  |  |
| NZ | 165 (80%) | 22 (79%) | 0.04, 0.85 |
| UK | 50 (77%) | 74 (85%) | 1.64, 0.20 |
| Brazil | 97 (84%) | 71 (83%) | 0.11, 0.74 |
| Ecuador | 41 (33%) | 21 (41%) | 1.11, 0.29 |
| Uganda | 52 (54%) | 51 (52%) | 0.09, 0.77 |
| **Blood eosinophils** absolute values (10^9^/L) median (range) |  |  |  |
| NZ | 0.40 (0 – 1.9) | 0.45 (0.1 – 1.1) | z=0.53, 0.59 |
| UK | 0.22 (0.02 – 0.81) | 0.19 (0.02 – 0.83) | z=-0.30, 0.77 |
| Brazil | 0.43 (0.04 – 1.23) | 0.35 (0.002 – 1.30) | z=-0.65, 0.52 |
| Ecuador | 0.56 (0.002 – 3.4) | 0.50 (0.04 – 1.85) | z=0.35, 0.73 |
| Uganda | 0.27 (0 – 2.99) | 0.22 (0.02 – 1.55) | z=-1.44, 0.15 |
| **FEV_1_ z-score** mean (SD) |  |  |  |
| NZ | -0.47 (1.03) | -0.58 (1.37) | t=-0.47, 0.64 |
| UK | -0.50 (1.35) | -0.33 (0.96) | t=0.93, 0.35 |
| Brazil | -0.73 (0.83) | -1.30 (1.16) | t=-4.03, 0.0001 |
| Ecuador | -0.23 (0.94) | -0.49 (1.01) | t=-1.54, 0.13 |
| Uganda | -0.23 (0.82) | -0.19 (0.98) | t=0.24, 0.81 |
| **Elevated FeNO level** n (%) |  |  |  |
| NZ | 96 (47%) | 15 (54%) | 0.48, 0.49 |
| Ecuador | 42 (34%) | 21 (42%) | 1.09, 0.30 |
| Uganda | 41 (43%) | 43 (42%) | 0.02, 0.89 |
| Controls | n=216 | n=140 |  |
| **Centre,** n (%) |  |  |  |
| NZ | 104 (79%) | 28 | 34.85, <0.001 |
| UK | 31 (46%) | 36 |  |
| Brazil | 20 (50%) | 20 |  |
| Ecuador | 41 (61%) | 26 |  |
| Uganda | 20 (40%) | 30 |  |
| **Age** (mean, range) |  |  |  |
| NZ | 14.9 (9.0 – 18.8) | 13.4 (9.2 – 19.8) | t=-2.36, 0.02 |
| UK | 25.9 (24.7 – 27.3) | 26.2 (24.6 – 27.3) | t=1.21, 0.23 |
| Brazil | 19.7 (17.3 – 23.0) | 19.9 (17.5 – 23.2) | t=0.27, 0.79 |
| Ecuador | 11.7 (11.0 – 12.1) | 11.6 (10.6 – 12.1) | t=-1.37, 0.18 |
| Uganda | 15.7 (13.0 – 18.9) | 15.2 (10.0 – 18.5) | t=-0.99, 0.33 |
| **Sex** n (%) female |  |  |  |
| NZ | 62 (60%) | 17 (61%) | 0.01, 0.92 |
| UK | 18 (58%) | 28 (78%) | 3.01, 0.08 |
| Brazil | 15 (75%) | 12 (60%) | 1.03, 0.31 |
| Ecuador | 11 (27%) | 12 (46%) | 2.64, 0.10 |
| Uganda | 12 (60%) | 16 (53%) | 0.22, 0.64 |
| **Skin prick test positive**  n (%) |  |  |  |
| NZ | 40 (39%) | 11 (42%) | 0.10, 0.75 |
| UK | 9 (31%) | 10 (28%) | 0.08, 0.77 |
| Brazil | 14 (70%) | 12 (60%) | 0.44, 0.51 |
| Ecuador | 7 (17%) | 2 (8%) | 1.09, 0.30 |
| Uganda | 3 (15%) | 3 (11%) | 0.16, 0.69 |
| **Blood eosinophils** absolute values (10^9^/L) median (range) |  |  |  |
| NZ | 0.20 (0.1 – 2.2) | 0.2 (0 – 1.1) | z=0.82, 0.41 |
| UK | 0.11 (0.02 – 0.51) | 0.08 (0.02 – 0.32) | z=-1.66, 0.10 |
| Brazil | 0.15 (0.04 – 2.71) | 0.19 (0 – 0.59) | z=0.00, 1.00 |
| Ecuador | 0.49 (0 – 1.88) | 0.37 (0 – 1.50) | z=-1.16, 0.24 |
| Uganda | 0.26 (0.05 – 0.81) | 0.12 (0.03 – 0.83) | z=-1.97, 0.05 |
| **FEV_1_ z-score** mean (SD) |  |  |  |
| NZ | -0.12 (0.99) | -0.07 (0.96) | t=0.24, 0.81 |
| UK | -0.19 (1.02) | -0.05 (1.16) | t=0.50, 0.62 |
| Brazil | -0.21 (0.94) | -0.49 (0.96) | t=-0.91, 0.37 |
| Ecuador | -0.002 (0.93) | 0.30 (1.22) | t=1.15, 0.26 |
| Uganda | -0.26 (1.04) | 0.26 (1.36) | t=1.25, 0.22 |
| **Elevated FeNO level** n (%) |  |  |  |
| NZ | 15 (15%) | 8 (30%) | 3.33, 0.07 |
| Ecuador | 9 (22%) | 5 (19%) | 0.07, 0.79 |
| Uganda | 2 (10%) | 3 (12%) | 0.03, 0.87 |

*In the past 12 months

**Table S2:** Association between centre and EA v paucigranulocytic, and neutrophilic v paucigranulocytic from multinomial regression. N=623

| Centre | EA (eosinophilic  + mixed) | Neutrophilic | Paucigranulocytic | Unadjusted  odds ratio (OR) (95% CI) | | Adjusted for age  OR (95% CI) | | Adjusted for age & sex  OR (95% CI) | |
| --- | --- | --- | --- | --- | --- | --- | --- | --- | --- |
|  |  |  |  | EA v Paucigranulocytic | Neutrophilic v Paucigranulocytic | EA v Paucigranulocytic | Neutrophilic v Paucigranulocytic | EA v Paucigranulocytic | Neutrophilic v Paucigranulocytic |
| NZ (baseline) | 104 (50%) | 14 (7%) | 89 (43%) | 1.0 | 1.0 | 1.0 | 1.0 | 1.0 | 1.0 |
| UK | 26 (33%) | 6 (8%) | 46 (59%) | 0.48 (0.28 – 0.85) | 0.83 (0.30 – 2.30) | 1.47 (0.51 – 0.25) | 11.85 (1.41 – 99.90) | 1.48 (0.51 – 4.31) | 12.15 (1.43 – 103.03) |
| Brazil | 40 (35%) | 5 (4%) | 70 (61%) | 0.49 (0.30 – 0.79) | 0.45 (0.16 – 1.32) | 0.70 (0.40 – 1.23) | 1.09 (0.32 – 3.77) | 0.76 (0.43 – 1.34) | 1.08 (0.31 – 3.76) |
| Ecuador | 40 (32%) | 8 (6%) | 77 (62%) | 0.44 (0.28 – 0.72) | 0.66 (0.26 – 1.66) | 0.36 (0.21 – 0.60) | 0.44 (0.17 – 1.13) | 0.35 (0.21 – 0.59) | 0.43 (0.17 – 1.13) |
| Uganda | 33 (34%) | 34 (35%) | 31 (32%) | 0.91 (0.52 – 1.60) | 6.97 (3.31 – 14.68) | 1.02 (0.57 – 1.82) | 9.86 (4.34 – 22.39) | 1.16 (0.65 – 2.08) | 9.69 (4.23 – 22.19) |
|  |  |  |  |  |  |  |  |  |  |
| Age (years) |  |  |  |  |  | 0.91 (0.84 – 0.98) | 0.80 (0.69 – 0.93) | 0.92 (0.85 – 0.99) | 0.80 (0.69 – 0.83) |
| Sex (female) |  |  |  |  |  |  |  | 0.54 (0.37 – 0.77) | 1.13 (0.60 – 2.14) |

**Table S3: Centre-specific participant and clinical characteristics by inflammatory phenotype**

**Brazil**

| **Inflammatory phenotype** | **eosinophilic** | **mixed granulocytic** | **neutrophilic** | **paucigranulocytic** | **Asthmatics** without sputum result | **Controls** (with sputum results) |
| --- | --- | --- | --- | --- | --- | --- |
|  | **n=38** | **n=2** | **n=5** | **n=70** | **n=89** | **n=20** |
| Female, n (%) | 24 (63%) | 2 (100%) | 4 (80%) | 51 (73%) | 53 (60%) | 15 (75%) |
| Age at questionnaire, years: mean (range) | 17.8 (12.5 – 23.8) | 18.3 (18.0 – 18.6) | 17.6 (16.3 – 19.0) | 18.4 (13.1 – 23.1) | 18.4 (12.0 – 23.9) | 19.7 (17.3 – 23.0) |
| Asthma diagnosis confirmed by doctor | 30 (79%) | 2 (100%) | 3 (60%) | 44 (63%) | 71 (80%) | - |
| Age at asthma diagnosis, years: median (range) | 7 (1 – 23) | 5 ( 5 – 5) | 2 (1 – 5) | 7 (1 – 20) | 5 (0 – 18) | - |
| missing | 11 | 1 | 2 | 29 | 26 |  |
| **Asthma severity *, n (%)** |  |  |  |  |  | - |
| mild or moderate | 6 (17%) | 0 (0%) | 4 (80%) | 20 (34%) | 32 (41%) |  |
| Severe | 30 (83%) | 2 (100%) | 1 (20%) | 38 (66%) | 47 (59%) |  |
| Missing | 2 | 0 | 0 | 12 | 10 |  |
| **Severe asthma** (>12 attacks*)**, n (%)** | 4 (11%) | 0 (0%) | 0 (0%) | 5 (7%) | 8 (9%) |  |
| **Asthma medication*, n (%)** |  |  |  |  |  | - |
| none | 8 (21%) | 0 (0%) | 3 (60%) | 23 (33%) | 21 (24%) |  |
| ICS (preventer inhaler) | 9 (24%) | 1 (50%) | 0 (0%) | 12 (17%) | 21 (24%) |  |
| Bronchodilator (reliever inhaler) | 22 (58%) | 1 (50%) | 2 (40%) | 42 (60%) | 57 (64%) |  |
| **ACQ score (past week)** |  |  |  |  |  | - |
| Median (IQR, range) | 0.92 (0.17 – 1.83, 0 – 2.83) | 1.83 (0.33 – 3.33, 0.33 – 3.33) | 0.17 (0.08 – 0.33, 0 – 0.5) | 1.0 (0.33 – 1.67, 0 – 3) | 0.5 (0.17 – 1.33, 0 – 4.17) |  |
| Well controlled (score<1.5) , n (%) | 25 (66%) | 1 (50%) | 4 (100%) | 46 (66%) | 68 (78%) |  |
| Not well controlled (score ≥1.5) , n (%) | 13 (34%) | 1 (50%) | 0 (0%) | 24 (34%) | 19 (22%) |  |
| missing | 0 | 0 | 1 | 0 | 2 |  |
| **Lung function** absolute values (L) & GLI-2012 z-scores | n=37 | n=2 | n=5 | n=68 | n=86 | n=19 |
| - FEV_1_, mean (SD) range | 2.98 (0.66)  1.97 – 4.48 | 2.23 (0.11)  2.15 – 2.31 | 3.20 (0.74)  2.44 – 4.33 | 2.92 (0.48)  1.64 – 3.99 | 2.84 (0.70)  1.44 – 4.84 | 3.35 (0.73)  2.50 – 4.73 |
| - FEV_1_ z-score, mean (SD) range | -0.80 (0.84)  -3.05 – 0.71 | -1.90 (0.62)  -2.34 – -1.46 | -0.46 (0.49)  -1.14 – 0.02 | -0.68 (0.84)  -2.75 – 1.36 | -1.30 (1.16)  -4.03 – 0.90 | -0.21 (0.94)  -1.49 – 1.99 |
| - FVC, mean (SD) range | 3.69 (0.85)  2.20 – 5.99 | 1.81 (1.96)  0.42 – 3.19 | 3.63 (0.78)  2.88 – 4.87 | 3.46 (0.62)  2.43 – 4.80 | 3.50 (0.98)  0.91 – 6.80 | 3.76 (0.80)  2.80 – 5.24 |
| - FVC z-score, mean (SD) range | -0.15 (0.99)  -2.64 – 1.63 | -3.63 (5.49)  -7.51 – 0.25 | -0.41 (0.49)  -1.02 – 0.20 | -0.37 (0.86)  -2.64 – 2.37 | -0.84 (1.32)  -5.95 – 2.01 | -0.33 (1.19)  -2.64 – 2.43 |
| - FEV_1_/FVC, mean (SD) range | 0.81 (0.09)  0.64 – 0.99 | 0.73 (0.13)  0.63 – 0.82 | 0.88 (0.03)  0.85 – 0.92 | 0.85 (0.09)  0.63 – 1.00 | 0.82 (0.12)  0.40 – 1.00 | 0.88 (0.06)  0.73 – 0.99 |
| - FEV_1_/FVC z-score, mean (SD) range | -1.04 (1.29)  -3.14 – 2.18 | -2.25 (1.22)  -3.12 – -1.39 | -0.19 (0.54)  -0.81 – 0.35 | -0.46 (1.28)  -3.18 – 2.23 | -0.79 (1.63)  -4.67 – 2.76 | 0.06 (1.11)  -2.40 – 1..87 |
| **Skin prick test positive, n (%)** | 37 (97%) | 1 (50%) | 4 (80%) | 55 (79%) | 71 (83%) | 14 (70%) |
| Not done | 0 | 0 | 0 | 0 | 3 | 0 |
| **Blood eosinophils** absolute values(10^9^/L) median (range) | n=36  0.51  (0.25 – 1.23) | n=2  0.47  (0.33 – 0.60) | n=5  0.13  (0.04 – 0.32) | n=66  0.33  (0.04 – 0.91) | n=87  0.35  (0.002 – 1.30) | n=20  0.15  (0.04 – 2.71) |

*In the past 12 months

**Ecuador**

| **Inflammatory phenotype** | **eosinophilic** | **mixed granulocytic** | **neutrophilic** | **paucigranulocytic** | **Asthmatics** without sputum result | **Controls** (with sputum results) |
| --- | --- | --- | --- | --- | --- | --- |
|  | **n=35** | **n=5** | **n=8** | **n=77** | **n=51** | **n=41** |
| Female, n (%) | 10 (29%) | 2 (40%) | 3 (38%) | 44 (57%) | 17 (33%) | 11 (27%) |
| Age at questionnaire, years: mean (range) | 12.4  (10.5 – 16.2) | 13.8  (11.7 – 16.8) | 12.3  (10.3 – 14.6) | 11.9  (10.3 – 16.9) | 11.8  (10.3 – 14.3) | 11.7  (11.0 – 12.1) |
| Asthma diagnosis confirmed by doctor | 29 (83%) | 5 (100%) | 5 (63%) | 40 (52%) | 29 (57%) | - |
| Age at asthma diagnosis, years: median (range) | 4 (0.08 – 9) | 4 (0.7 – 13) | 1.3 (0.3 – 6) | 2 (0 – 14) | 2 (0 – 10) | - |
| **Asthma severity *, n (%)** |  |  |  |  |  | - |
| mild or moderate | 20 (57%) | 3 (60%) | 4 (50%) | 45 (59%) | 24 (47%) |  |
| Severe | 15 (43%) | 2 (40%) | 4 (50%) | 31 (41%) | 27 (53%) |  |
| Missing | 0 | 0 | 0 | 1 | 0 |  |
| **Severe asthma** (>12 attacks*)**, n (%)** | 0 | 0 | 0 | 0 | 0 |  |
| **Asthma medication*, n (%)** |  |  |  |  |  | - |
| None | 13 (37%) | 1 (20%) | 5 (63%) | 52 (68%) | 28 (55%) |  |
| ICS (preventer inhaler) | 0 (0%) | 0 (0%) | 0 (0%) | 6 (8%) | 0 (0%) |  |
| Bronchodilator (reliever inhaler) | 7 (20%) | 1 (20%) | 0 (0%) | 10 (13%) | 9 (18%) |  |
| **ACQ score (past week)** |  |  |  |  |  |  |
| Median (IQR, range) | 0 (0 – 0, 0 – 2.67) | 0 (0 – 0, 0 – 2) | 0 (0 – 0, 0 – 0) | 0 (0 – 0, 0 – 1.5) | 0 (0 – 0, 0 – 2.67) | - |
| Well controlled (score<1.5), n (%) | 32 (91%) | 4 (80%) | 8 (100%) | 76 (99%) | 48 (94%) |  |
| Not well controlled (score ≥1.5), n (%) | 3 (9%) | 1 (20%) | 0 (0%) | 1 (1%) | 3 (6%) |  |
| **Lung function** absolute values (L) & GLI-2012 z-scores | n=34 | n=5 | n=8 | n=77 | n=48 | n=41 |
| - FEV_1_, mean (SD) range | 2.11 (0.56)  1.16 – 3.75 | 2.36 (0.52)  1.62 – 3.00 | 2.03 (0.49)  1.58 – 2.86 | 2.15 (0.43)  1.37 – 3.79 | 2.00 (0.39)  1.31 – 2.88 | 2.05 (0.35)  1.35 – 3.19 |
| - FEV_1_ z-score, mean (SD) range | -0.48 (0.96)  -3.03 – 1.26 | -0.63 (1.07)  -2.11 – 0.70 | -0.50 (0.80)  -1.37 – 0.97 | -0.07 (0.92)  -2.31 – 2.07 | -0.49 (1.01)  -2.46 – 2.17 | -0.002 (0.93)  -1.99 – 2.80 |
| - FVC, mean (SD) range | 2.39 (0.65)  1.28 – 4.21 | 2.58 (0.52)  1.76 – 3.07 | 2.25 (0.61)  1.69 – 3.38 | 2.36 (0.46)  1.49 – 4.23 | 2.25 (0.48)  1.34 – 3.29 | 2.25 (0.38)  1.57 – 3.46 |
| - FVC z-score, mean (SD) range | -0.56 (0.95)  -3.21 – 1.45 | -0.86 (1.29)  -2.25 – 0.45 | -0.69 (1.10)  -1.78 – 1.01 | -0.33 (0.94)  -2.75 – 1.53 | -0.62 (1.11)  -3.30 – 2.71 | -0.34 (0.94)  -2.25 – 2.07 |
| - FEV_1_/FVC, mean (SD) range | 0.89 (0.06)  0.75 – 1.00 | 0.91 (0.06)  0.85 – 1.00 | 0.91 (0.03)  0.85 – 0.93 | 0.91 (0.05)  0.74 – 1.00 | 0.89 (0.06)  0.77 – 0.99 | 0.91 (0.05)  0.81 – 0.99 |
| - FEV_1_/FVC z-score, mean (SD) range | 0.16 (1.07)  -1.91 – 2.29 | 0.38 (1.22)  -0.87 – 2.29 | 0.35 (0.68)  -0.99 – 1.19 | 0.54 (1.01)  -2.34 – 2.61 | 0.26 (1.11)  -1.76 – 2.47 | 0.67 (0.96)  -1.20 – 2.46 |
| **FeNO level, n (%)** |  |  |  |  |  |  |
| normal | 12 | 3 | 8 | 60 | 29 | 32 |
| elevated | 23 (66%) | 2 (40%) | 0 (0%) | 17 (22%) | 21 (42%) | 9 (22%) |
| not measured | 0 | 0 | 0 | 0 | 1 | 0 |
| **Skin prick test positive, n (%)** | 21 (60%) | 2 (40%) | 2 (25%) | 16 (21%) | 21 (41%) | 7 (17%) |
| **Blood eosinophils** absolute values (10^9^/L) median (range) | n=35  0.65  (0.002 – 2.74) | n=5  0.70  (0.19 – 1.18) | n=8  0.40  (0.11 – 1.68) | n=77  0.48  (0.07 – 3.40) | n=51  0.50  (0.04 – 1.85) | n=41  0.49  (0 – 1.88) |

*In the past 12 months

**New Zealand**

| **Inflammatory phenotype** | **eosinophilic** | **mixed granulocytic** | **neutrophilic** | **paucigranulocytic** | **Asthmatics** without sputum result | **Controls** (with sputum results) |
| --- | --- | --- | --- | --- | --- | --- |
|  | n=**99** | n=**5** | n=**14** | n=**89** | n=**28** | n=**104** |
| Female, n (%) | 45 (45%) | 2 (40%) | 10 (71%) | 46 (52%) | 10 (36%) | 62 (60%) |
| Age at questionnaire, years: mean (range) | 14.0 (9.1 – 20.0) | 11.8 (8.6 – 16.0) | 11.8 (8.8 – 15.7) | 15.1 (9.3 – 20.3) | 12.6 (9.0 – 17.9) | 14.9 (9.0 – 18.8) |
| Asthma diagnosis confirmed by doctor | 94 (95%) | 5 (100%) | 13 (93%) | 77 (87%) | 25 (89%) | - |
| Age at asthma diagnosis, years: median (range) | 3 (0 – 13) | 2 (1 – 4) | 4 (1 – 8) | 5 (1 – 16) | 3 (1 – 11) | - |
| missing | 20 | 0 | 2 | 19 | 4 |  |
| **Asthma severity *, n (%)** |  |  |  |  |  | - |
| mild or moderate | 45 | 1 | 7 | 56 | 14 |  |
| severe | 54 (55%) | 4 (80%) | 7 (50%) | 33 (37%) | 14 (50%) |  |
| missing |  |  |  |  |  |  |
| **Severe asthma** (>12 attacks*), n (%) | 18 (18%) | 1 (20%) | 2 (14%) | 8 (9%) | 7 (25%) |  |
| **Asthma medication*, n(%)** |  |  |  |  |  | - |
| None | 2 (2%) | 0 (0%) | 0 (0%) | 11 (12%) | 1 (4%) |  |
| ICS (preventer inhaler) | 72 (73%) | 5 (100%) | 9 (64%) | 56 (63%) | 20 (71%) |  |
| Bronchodilator (reliever inhaler) | 91 (92%) | 5 (100%) | 14 (100%) | 74 (83%) | 26 (93%) |  |
| **ACQ score (past week)** |  |  |  |  |  | - |
| Median (IQR, range) |  |  |  |  |  |  |
| Well controlled (score<1.5), n (%) | 67 (76%) | 2 (40%) | 14 (100%) | 66 (84%) | 21 (88%) |  |
| Not well controlled (score ≥1.5) , n (%) | 21 (24%) | 3 (60%) | 0 (0%) | 13 (16%) | 3 (12%) |  |
| Missing | 11 | 0 | 0 | 10 | 4 |  |
| **Lung function** absolute values (L) & GLI-2012 z-scores | n=99 | n=5 | n=14 | n=89 | n=27 | n=104 |
| - FEV_1_, mean (SD) range | 2.65 (0.88)  1.13 – 4.84 | 1.78 (0.44)  1.20 – 2.11 | 2.14 (0.61)  1.46 – 3.34 | 3.18 (0.94)  1.36 – 5.37 | 2.29 (0.75)  1.41 – 4.27 | 3.12 (0.97)  1.31 – 5.42 |
| - FEV_1_ z-score, mean (SD) range | -0.72 (0.98)  -2.95 – 1.61 | -0.87 (1.77)  -3.52 – 0.89 | -0.28 (1.07)  -1.94 – 1.51 | -0.21 (0.97)  -2.24 – 2.36 | -0.58 (1.37)  -3.08 – 2.13 | -0.12 (0.99)  -2.41 – 2.60 |
| - FVC, mean (SD) range | 3.30 (1.10)  1.62 – 6.05 | 2.45 (0.81)  1.36 – 3.62 | 2.56 (0.69)  1.80 – 3.64 | 3.81 (1.09)  1.81 – 6.05 | 2.94 (1.09)  1.79 – 5.78 | 3.63 (1.14)  1.44 – 6.31 |
| - FVC z-score, mean (SD) range | -0.07 (0.95)  -2.15 – 2.15 | 0.35 (1.17)  -1.42 – 1.24 | 0.23 (1.35)  -2.07 – 2.58 | 0.19 (0.86)  -2.56 – 2.57 | 0.19 (1.21)  -1.62 – 2.97 | -0.01 (0.89)  -2.16 – 2.28 |
| - FEV_1_/FVC, mean (SD) range | 0.82 (0.07)  0.59 – 0.99 | 0.76 (0.13)  0.60 – 0.88 | 0.83 (0.05)  0.77 – 0.92 | 0.84 (0.07)  0.68 – 0.99 | 0.79 (0.07)  0.62 – 0.90 | 0.87 (0.06)  0.67 – 0.99 |
| - FEV_1_/FVC z-score, mean (SD) range | -0.94 (0.99)  -3.62 – 2.29 | -1.81 (1.49)  -3.59 – -0.51 | -0.87 (0.75)  -1.50 – 0.51 | -0.49 (1.07)  -2.70 – 2.28 | -1.25 (0.97)  -3.00 – 0.69 | -0.11 (0.89)  -2.68 – 2.14 |
| **FeNO level, n (%)** |  |  |  |  |  |  |
| Normal | 31 | 2 | 8 | 69 | 13 | 88 |
| Elevated | 68 (69%) | 3 (60%) | 6 (43%) | 19 (22%) | 15 (54%) | 15 (15%) |
| not measured | 0 | 0 | 0 | 1 | 0 | 1 |
| **Skin prick test positive, n (%)** | 83 (85%) | 4 (80%) | 11 (79%) | 67 (75%) | 22 (79%) | 40 (39%) |
| Not done | 1 | 0 | 0 | 0 | 0 | 1 |
| **Blood eosinophils** absolute values (10^9^/L) median (range) | n=73  0.60 (0 – 1.90) | n=4  0.60 (0.10 – 1.00) | n=9  0.50 (0.10 – 0.70) | n=77  0.30 (0 – 1.90) | n=20  0.45 (0.10 – 1.10) | n=88  0.20 (0.10 – 2.20) |

*In the past 12 months

**Uganda**

| **Inflammatory phenotype** | **eosinophilic** | **mixed granulocytic** | **neutrophilic** | **paucigranulocytic** | **Asthmatics** without sputum result | **Controls** (with sputum results) |
| --- | --- | --- | --- | --- | --- | --- |
|  | **n=25** | **n=8** | **n=34** | **n=31** | **n=109** | **n=20** |
| Female, n (%) | 14 (56%) | 6 (75%) | 26 (76%) | 27 (87%) | 82 (75%) | 12 (60%) |
| Age at questionnaire, years: mean (range) | 15.2 (12.7 – 17.9) | 15.7 (13.0 – 17.5) | 15.5 (12.0 – 17.8) | 15.6 (12.1 – 17.9) | 15.7 (12.2 – 18.0) | 15.7 (13.0 – 18.9) |
| Asthma diagnosis confirmed by doctor | 17 (68%) | 6 (75%) | 16 (47%) | 19 (61%) | 83 (76%) | - |
| Age at asthma diagnosis, years: median (range) | 9 (1-13) | 5 (1-13) | 8 (1-16) | 12 (1-15) | 8 (1-17 ) | - |
| **Asthma severity *, n (%)** |  |  |  |  |  | - |
| mild or moderate | 9 | 2 | 18 | 14 | 28 |  |
| Severe | 16 (64%) | 6 (75%) | 16 (47%) | 17 (55%) | 81 (74%) |  |
| **Severe asthma** (>12 attacks*), n (%) | 5 (20%) | 1 (13%) | 6 (18%) | 5 (16%) | 22 (20%) |  |
| **Asthma medication*, n (%)** |  |  |  |  |  | - |
| none | 6 (24%) | 2 (25%) | 12 (35%) | 10 (32%) | 25 (23%) |  |
| ICS (preventer inhaler) | 4 (16%) | 2 (25%) | 2 (6%) | 3 (10%) | 22 (20%) |  |
| Bronchodilator (reliever inhaler) | 8 (32%) | 5 (63%) | 6 (18%) | 12 (39%) | 51 (47%) |  |
| **ACQ score (past week)** |  |  |  |  |  |  |
| Median (IQR, range) | 0.5, 0 – 1.67, 0 – 2.67 | 1.0, 0 – 1.92, 0 – 2.5 | 1.1, 0.17 – 1.5, 0 – 4 | 0.33, 0 – 1.5, 0 - 3 | 0.42, 0 – 1.6, 0 – 5 | - |
| Well controlled (score<1.5), n (%) | 17 (74%) | 5 (63%) | 22 (65%) | 22 (71%) | 53 (74%) |  |
| Not well controlled (score ≥1.5) , n (%) | 6 (26%) | 3 (37%) | 12 (35%) | 9 (29%) | 19 (26%) |  |
| Not done | 2 | 0 | 0 | 0 | 37 |  |
| **Lung function** absolute values (L) & GLI-2012 z-scores | n=17 | n=7 | n=27 | n=24 | n=63 | n=16 |
| - FEV_1_, mean (SD) range | 2.54 (0.60)  1.81 – 3.98 | 2.57 (1.00)  1.73 – 4.02 | 2.53 (0.35)  1.95 – 3.14 | 2.54 (0.38)  2.04 – 3.38 | 2.67 (0.45)  1.68 – 4.06 | 2.47 (0.63)  1.57 – 3.72 |
| - FEV_1_ z-score, mean (SD) range | -0.40 (0.81)  -1.48 – 1.66 | -0.18 (1.35)  -1.58 – 1.89 | -0.29 (0.76)  -1.50 – 1.03 | -0.06 (0.70)  -1.23 – 1.30 | -0.19 (0.98)  -2.78 – 2.23 | -0.26 (1.04)  -1.97 – 1.60 |
| - FVC, mean (SD) range | 2.99 (0.55)  2.34 – 4.13 | 3.55 (2.19)  2.07 – 8.01 | 2.91 (0.35)  2.25 – 3.55 | 2.92 (0.53)  2.15 – 4.57 | 3.12 (0.58)  1.77 – 5.01 | 2.89 (0.78)  1.65 – 4.53 |
| - FVC z-score, mean (SD) range | -0.03 (0.88)  -1.31 – 1.64 | 1.22 (2.91)  -1.11 – 7.14 | -0.11 (0.78)  -1.72 – 1.13 | 0.17 (1.07)  -1.82 – 2.98 | 0.07 (1.04)  -1.94 – 3.05 | 0.11 (1.52)  -2.34 – 4.13 |
| - FEV_1_/FVC, mean (SD) range | 0.85 (0.09)  0.69 – 0.99 | 0.79 (0.14)  0.49 – 0.91 | 0.87 (0.06)  0.74 – 0.97 | 0.88 (0.08)  0.67 – 0.99 | 0.86 (0.09)  0.63 – 1.00 | 0.87 (0.12)  0.48 – 1.00 |
| - FEV_1_/FVC z-score, mean (SD) range | -0.55 (1.41)  -2.69 – 1.98 | -1.45 (1.38)  -4.25 - -0.04 | -0.34 (1.00)  -2.13 – 1.46 | -0.25 (1.21)  -2.90 – 1.78 | -0.35 (1.37)  -3.14 – 2.51 | -0.26 (1.46)  -3.92 – 1.93 |
| **FeNO level, n (%)** |  |  |  |  |  |  |
| normal | 6 | 2 | 22 | 24 | 59 | 18 |
| elevated | 17 (74%) | 6 (75%) | 12 (35%) | 6 (20%) | 43 (42%) | 2 (10%) |
| not measured | 2 | 0 | 0 | 1 | 7 | 0 |
| **Skin prick test positive, n (%)** | 21 (84%) | 4 (50%) | 18 (53%) | 9 (30%) | 51 (52%) | 3 (15%) |
| Not tested | 0 | 0 | 0 | 1 | 10 | 0 |
| **Blood eosinophils** absolute values (10^9^/L) median (range) | n=25  0.47  (0.07 – 2.99) | n=8  0.31  (0.19 – 0.80) | n=34  0.22  (0.01 – 2.95) | n=31  0.23  (0 – 1.09) | n=98  0.22  (0.02 – 1.55) | n=20  0.26  (0.05 – 0.81) |

*In the past 12 months

**United Kingdom**

| **Inflammatory phenotype** | **eosinophilic** | **mixed granulocytic** | **neutrophilic** | **paucigranulocytic** | **Asthmatics** without sputum result | **Controls** (with sputum results) |
| --- | --- | --- | --- | --- | --- | --- |
|  | **n=24** | **n=2** | **n=6** | **n=46** | **n=98** | **n=31** |
| Female, n (%) | 14 (58%) | 2 (100%) | 5 (83%) | 37 (80%) | 73 (74%) | 18 (58%) |
| Age at questionnaire, years: mean (range) | 25.9 (25.0 – 26.6) | 25.8 (25.6 – 26.1) | 26.0 (25.3 – 26.7) | 25.9 (24.9 – 27.2) | 25.9 (24.6 – 27.3) | 25.9 (24.7 – 27.3) |
| Asthma diagnosis confirmed by doctor | 23 (96%) | 2 (100%) | 6 (100%) | 45 (98%) | 95 (97%) | - |
| **Asthma severity *, n (%)** |  |  |  |  |  | - |
| mild or moderate | 7 | 1 | 5 | 30 | 54 |  |
| severe | 17 (71%) | 1 (50%) | 1 (17%) | 16 (35%) | 44 (45%) |  |
| **Severe asthma** (>12 attacks*), n (%) | 9 (38%) | 1 (50%) | 0 (0%) | 3 (7%) | 14 (14%) |  |
| **Asthma medication*, n (%)** |  |  |  |  |  | - |
| none | 2 (8%) | 0 (0%) | 0 (0%) | 5 (11%) | 20 (20%) |  |
| ICS (preventer inhaler) | 17 (71%) | 2 (100%) | 5 (83%) | 26 (57%) | 44 (45%) |  |
| Bronchodilator (reliever inhaler) | 22 (92%) | 2 (100%) | 6 (100%) | 40 (87%) | 75 (77%) |  |
| **ACQ score (past week)** |  |  |  |  |  | - |
| Median (IQR, range) | 1.0 (0.50 – 1.33, 0 – 3) | 1.0 (0.83 – 1.17, 0.83 – 1.17) | 0.25 (0 – 0.67, 0 – 1.33) | 0.33 (0 – 1.0, 0 – 2.5) | 0.17 (0 – 0.83, 0 – 2.83) |  |
| Well controlled (score<1.5), n (%) | 19 (83%) | 2 (100%) | 6 (100%) | 39 (87%) | 88 (93%) |  |
| Not well controlled (score ≥1.5), n (%) | 4 (17%) | 0 | 0 | 6 (13%) | 7 (7%) |  |
| Not done | 1 | 0 | 0 | 1 | 3 |  |
| **Lung function** absolute values (L) & GLI-2012 z-scores | N=24 | N=2 | N=5 | N=44 | N=95 | N=31 |
| - FEV_1_, mean (SD) range | 3.67 (0.90)  2.39 – 5.45 | 2.95 (0.95)  2.28 – 3.62 | 3.77 (0.75)  2.97 – 4.62 | 3.33 (0.78)  1.62 – 5.29 | 3.51 (0.74)  2.36 – 5.61 | 3.97 (0.91)  2.59 – 6.32 |
| - FEV_1_ z-score, mean (SD) range | -0.45 (1.04)  -3.02 – 0.96 | -0.88 (3.02)  -3.02 – 1.26 | -0.07 (1.60)  -1.64 – 2.43 | -0.56 (1.43)  -4.12 – 3.59 | -0.33 (0.96)  -2.52 – 2.82 | -0.19 (1.02)  -2.62 – 2.76 |
| - FVC, mean (SD) range | 4.80 (1.24)  3.33 – 7.78 | 4.07 (1.24)  3.19 – 4.95 | 4.53 (0.82)  3.65 – 5.69 | 4.05 (0.95)  1.80 – 6.34 | 4.25 (1.02)  2.78 – 7.45 | 4.79 (1.33)  3.01 – 8.11 |
| - FVC z-score, mean (SD) range | 0.34 (0.85)  -1.62 – 1.96 | 0.35 (3.33)  -2.00 – 2.70 | 0.02 (1.26)  -1.61 – 1.67 | -0.33 (1.32)  -4.52 – 3.74 | -0.14 (0.89)  -2.03 – 1.65 | -0.11 (1.09)  -2.12 – 3.12 |
| - FEV_1_/FVC, mean (SD) range | 0.78 (0.10)  0.47 – 0.92 | 0.72 (0.01)  0.71 – 0.73 | 0.83 (0.04)  0.80 – 0.90 | 0.83 (0.07)  0.67 – 0.95 | 0.83 (0.07)  0.65 – 0.97 | 0.84 (0.07)  0.70 – 0.97 |
| - FEV_1_/FVC z-score, mean (SD) range | -1.01 (1.25)  -4.05 – 1.00 | -1.90 (0.11)  -1.97 – -1.82 | -0.33 (0.64)  -0.89 – 0.73 | -0.42 (1.00)  -2.73 – 1.71 | -0.30 (1.00)  -2.63 – 2.20 | -0.12 (0.92)  -2.11 – 1.96 |
| **Skin prick test positive, n (%)** | 18 (95%) | 2 (100%) | 3 (50%) | 27 (71%) | 74 (85%) | 9 (31%) |
| Not tested | 5 | 0 | 0 | 8 | 11 | 2 |
| **Blood eosinophils** absolute values (10^9^/L) median (range) | n=20  0.39 (0.11 – 0.81) | n=2  0.22 (0.06 – 0.37) | n=5  0.14 (0.09 – 0.56) | n=35  0.14 (0.02 – 0.41) | n=63  0.19 (0.02 – 0.83) | n=27  0.11 (0.02 – 0.51) |

*In the past 12 months

**Table S4: Comparison of sputum slide results, excluding low quality slides***

| **Centre** | **Brazil** | **Brazil excluding low quality slides*** | **Ecuador** | **Ecuador excluding low quality slides*** | **New Zealand** | **New Zealand, excluding low quality slides*** | **Uganda** | **Uganda excluding low quality slides*** | **United Kingdom** | **United Kingdom**  **excluding low quality slides*** |
| --- | --- | --- | --- | --- | --- | --- | --- | --- | --- | --- |
| **Asthma** **cases** | N=115 | N=87 | N=125 | N=111 | N=207 | N=116 | N=98 | N=75 | N=78 | N=50 |
| Sputum inflammatory phenotype, n (%) |  |  |  |  |  |  |  |  |  |  |
| eosinophilic | 38 (33%) | 26 (30%) | 35 (28%) | 31 (28%) | 99 (48%) | 57 (49%) | 25 (25%) | 15 (20%) | 24 (31%) | 14 (28%) |
| mixed granulocytic | 2 (2%) | 2 (2%) | 5 (4%) | 5 (5%) | 5 (2%) | 3 (3%) | 8 (8%) | 7 (9%) | 2 (3%) | 2 (4%) |
| neutrophilic | 5 (4%) | 5 (6%) | 8 (6%) | 7 (6%) | 14 (7%) | 11 (9%) | 34 (35%) | 30 (40%) | 6 (8%) | 6 (12%) |
| paucigranulocytic | 70 (61%) | 54 (62%) | 77 (62%) | 68 (61%) | 89 (43%) | 45 (39%) | 31 (32%) | 23 (31%) | 46 (59%) | 28 (56%) |
| **Repeat sputum slide, n (%)** |  |  |  |  |  |  |  |  |  |  |
| same phenotype (EA or NEA**) | 27 (68%) | 22 (69%) | 25 (69%) | 24 (69%) | 72 (67%) | 41 (64%) | 9 (75%) | 9 (90%) | 6 (55%) | 3 (60%) |
| Changed: |  |  |  |  |  |  |  |  |  |  |
| EA to NEA | 6 | 6 | 4 | 4 | 18 | 12 | 0 | 0 | 4 | 1 |
| NEA to EA | 7 | 4 | 7 | 7 | 17 | 11 | 3 | 1 | 1 | 1 |
| **Controls** | N=20 | N=11 | N=41 | N=39 | N=104 | N=64 | N=20 | N=17 | N=31 | N=22 |
| eosinophilic | 4 (20%) | 2 (18%) | 3 (7%) | 3 (8%) | 11 (11%) | 8 (12%) | 2 (10%) | 1 (6%) | 3 (10%) | 2 (9%) |
| mixed granulocytic | 0 | 0 | 0 | 0 | 1 (1%) | 1 (2%) | 1 (5%) | 1 (6%) | 0 | 0 |
| neutrophilic | 4 (20%) | 3 (27%) | 1 (2%) | 1 (2%) | 11 (11%) | 6 (9%) | 12 (60%) | 10 (59%) | 3 (10%) | 3 (14%) |
| paucigranulocytic | 12 (60%) | 6 (55%) | 37 (90%) | 35 (90%) | 81 (78%) | 49 (77%) | 5 (25%) | 5 (29%) | 25 (81%) | 17 (77%) |

*<400 total non-squamous cells and ≥30% squamous cells. ** EA (eosinophilic or mixed); NEA (neutrophilic or paucigranulocytic)

**Table S5** Sputum slide results with original and alternative phenotype definitions: A) 1% cut-off for eosinophils^1^ and B) 54% cut-off for neutrophils^2^

| **Centre** | **Brazil** | **Brazil A** | **Brazil B** | **Ecuador** | **Ecuador A** | **Ecuador B** | **NZ** | **NZ**  **A** | **NZ**  **B** | **Uganda** | **Uganda**  **A** | **Uganda**  **B** | **UK** | **UK**  **A** | **UK**  **B** |
| --- | --- | --- | --- | --- | --- | --- | --- | --- | --- | --- | --- | --- | --- | --- | --- |
| **Asthma** **cases** | N=115 | N=115 | N=115 | N=125 | N=125 | N=125 | N=207 | N=207 | N=207 | N=98 | N=98 | N=98 | N=78 | N=78 | N=78 |
| Sputum inflammatory phenotype, n(%) |  |  |  |  |  |  |  |  |  |  |  |  |  |  |  |
| eosinophilic | 38 (33%) | 56 (49%) | 38 (33%) | 35 (28%) | 43 (34%) | 35 (28%) | 99 (48%) | 130 (63%) | 94 (45%) | 25 (25%) | 30 (31%) | 24 (24%) | 24 (31%) | 39 (50%) | 23 (29%) |
| mixed granulocytic | 2 (2%) | 2 (2%) | 2 (2%) | 5 (4%) | 7 (6%) | 5 (4%) | 5 (2%) | 12 (6%) | 10 (5%) | 8 (8%) | 16 (16%) | 9 (9%) | 2 (3%) | 4 (5%) | 3 (4%) |
| neutrophilic | 5 (4%) | 5 (4%) | 8 (7%) | 8 (6%) | 6 (5%) | 9 (7%) | 14 (7%) | 7 (3%) | 17 (8%) | 34 (35%) | 26 (27%) | 39 (40%) | 6 (8%) | 4 (5%) | 11 (14%) |
| paucigranulocytic | 70 (61%) | 52 (45%) | 67 (58%) | 77 (62%) | 69 (55%) | 76 (61%) | 89 (43%) | 58 (28%) | 86 (42%) | 31 (32%) | 26 (27%) | 26 (27%) | 46 (59%) | 31 (40%) | 41 (53%) |
| **Repeat sputum slide, n(%)** |  |  |  |  |  |  |  |  |  |  |  |  |  |  |  |
| same phenotype (EA or NEA**) | 27 (68%) | 29 (73%) | 27 (68%) | 25 (69%) | 29 (81%) | 25 (69%) | 72 (67%) | 72 (67%) | 72 (67%) | 9 (75%) | 10 (83%) | 9 (75%) | 6 (55%) | 5 (45%) | 6 (55%) |
| Changed: |  |  |  |  |  |  |  |  |  |  |  |  |  |  |  |
| EA to NEA | 6 | 4 | 6 | 4 | 2 | 4 | 18 | 19 | 18 | 0 | 0 | 0 | 4 | 4 | 4 |
| NEA to EA | 7 | 7 | 7 | 7 | 5 | 7 | 17 | 16 | 17 | 3 | 2 | 3 | 1 | 2 | 1 |
| **Controls** | N=20 | N=20 | N=20 | N=41 | N=41 | N=41 | N=104 | N=104 | N=104 | N=20 | N=20 | N=20 | N=31 | N=31 | N=31 |
| eosinophilic | 4 (20%) | 5 (25%) | 4 (20%) | 3 (7%) | 8 (20%) | 3 (7%) | 11 (11%) | 24 (23%) | 10 (10%) | 2 (10%) | 2 (10%) | 2 (10%) | 3 (10%) | 6 (19%) | 3 (10%) |
| mixed granulocytic | 0 | 0 | 0 | 0 | 0 | 0 | 1 (1%) | 1 (1%) | 2 (2%) | 1 (5%) | 6 (30%) | 1 (5%) | 0 | 0 | 0 |
| neutrophilic | 4 (20%) | 4 (20%) | 5 (25%) | 1 (2%) | 1 (2%) | 1 (2%) | 11 (11%) | 11 (11%) | 17 (16%) | 12 (60%) | 7 (35%) | 13 (65%) | 3 (10%) | 3 (10%) | 3 (10%) |
| paucigranulocytic | 12 (60%) | 11 (55%) | 11 (55)% | 37 (90%) | 32 (78%) | 37 (90%) | 81 (78%) | 68 (65%) | 75 (72%) | 5 (25%) | 5 (25%) | 4 (20%) | 25 (81%) | 22 (71%) | 25 (81%) |

^1^ eosinophilic: ≥1% eosinophils; neutrophilic: <1% eosinophils and ≥61% neutrophils; mixed granulocytic: ≥1% eosinophils and ≥61% neutrophils; paucigranulocytic: <1% eosinophils and <61% neutrophils.

^2^ eosinophilic: ≥2.5% eosinophils; neutrophilic: <2.5% eosinophils and ≥54% neutrophils; mixed granulocytic: ≥2.5% eosinophils and ≥54% neutrophils; paucigranulocytic: <2.5% eosinophils and <54% neutrophils.
